# Long-term home-based physical exercise, pain, and use of pain medication over a year after hip fracture – A secondary analysis of a randomised controlled trial

**DOI:** 10.1177/02692155251389435

**Published:** 2025-10-23

**Authors:** Sara Suikkanen, Paula Soukkio, Mirjami Kantola, Hannu Kautiainen, Maija Haanpää, Markku T Hupli, Katriina Kukkonen-Harjula

**Affiliations:** 1 Faculty of Health Care and Social Services, 4414 LAB University of Applied Sciences, Lappeenranta, Finland; 2 Development Services, Wellbeing Services County of South Karelia, Lappeenranta, Finland; 3 Faculty of Sport and Health Sciences, 541605 University of Jyväskylä, Jyväskylä, Finland; 4 Primary Health Care Unit, 60650 Kuopio University Hospital, Kuopio, Finland; 5 Folkhälsan Research Center, Helsinki, Finland; 6 Ilmarinen Mutual Pension Insurance Company, Helsinki, Finland; 7 Department of Neurosurgery, Helsinki University Hospital, Helsinki, Finland; 8 743742 Mehiläinen, Lappeenranta, Finland; 9 Faculty of Medicine and Health Technology, 7840 Tampere University, Tampere, Finland

**Keywords:** Randomised controlled trial, rehabilitation, hip fracture, older adults, pain

## Abstract

**Objective:**

To study the effects of a year-long, supervised home-based exercise training on perceived pain, pain interference, and use of pain medication over 12 months after hip fracture.

**Design:**

Randomised clinical trial, secondary analysis

**Setting:**

Home

**Participants:**

Participants (*n* = 121) had surgical repair of a hip fracture, were ≥60 years old, and community-living.

**Intervention:**

Participants were allocated into 12-month home-based Physical Exercise (*n* = 61) or Usual Care (*n* = 60). Exercise sessions (60 minutes/twice a week) at participants’ home under physiotherapist supervision including strength, balance, and functional exercises.

**Main measures:**

Pain intensity, interference, and locations, and information of the pain medication were queried at baseline, 3, 6 and 12 months.

**Results:**

The mean age was 81 (SD 7) years, 91 (75%) were women, and 74 (61%) had fractured femoral neck. At baseline, in Physical Exercise 46 (75%) and in Usual Care 43 (72%) reported some sort of pain. After discharge, 118 (98%) used pain medication: 116 (96%) paracetamol and 41 (34%) opioids. At 12 months, there was no difference between groups in global pain prevalence, or in pain intensity, but the prevalence of hip pain (*P* = .047, effect size −0.38 (95% CI −0.51 to −0.22)) and pain interference (*P* = .042, effect size −0.18 (95% CI −0.52 to −0.05)) were lower in Physical Exercise than in Usual Care. At 12 months, there was no difference in medication use between the groups.

**Conclusion:**

The year-long supervised home-exercise reduced pain interference, and the prevalence of hip pain compared to usual care. Over 12 months the use of pain medication decreased in both groups.

**Registration:**

ClinicalTrials.gov (NCT02305433)

## Introduction

Hip fracture is a major trauma, which causes medium to long-term problems for older adults by decreasing functional capacity and quality of life.^1^ Excess mortality risk in patients with a hip fracture is higher than among those without one, even after a decade.^2^ Compared to age-matched controls, patients with hip fractures have poorer mobility, functional capacity, and quality of life, and more often increased need for 24-hour care.^1^ Even after rehabilitation, a person may not recover to a pre-fracture functional state.^3^

The main categories of hip fracture are intra-capsular femoral neck and extra-capsular basi-cervical, trochanteric and sub-trochanteric fractures.^4^ Operation of the femoral neck or trochanteric fracture is an invasive surgical operation that includes an internal fixation by osteosynthesis with, e.g. dynamic hip screws, intramedullary hip screws or parallel screws, or replacement of the femoral neck with prosthesis such as hemiarthroplasty.^4–7^ To decrease the possibility of complications after a hip fracture surgery, interdisciplinary care including professionals such as orthopaedic surgeons, geriatricians, nurses, and physical and occupational therapists^8,9^ is strongly recommended.^10^

As hip fracture surgery is a large invasive operation, it causes moderate-to-severe postoperative pain. During the operation, a single injection of femoral nerve or fascia iliac compartment block is recommended for the pain.^11^ In the hospital paracetamol, Non-Steroidal Anti-Inflammatory Drugs or cyclooxygenase-two inhibitors with opioids are recommended.^11^ Concerning pain medication recommendations for older adults, paracetamol is recommended for mild pain, as it is well tolerated and has fewer adverse effects than other pain medications. Non-Steroidal Anti-Inflammatory Drugs or weaker opioid agonists can be considered for mild to moderate pain, and pure opioid agonists for severe pain.^12^ Non-Steroidal Anti-Inflammatory Drugs, however, have many adverse effects, such as gastrointestinal toxicity (e.g. gastroduodenal ulcers), cardiovascular (e.g. oedema, hypertension, myocardial infarction, congestive heart failure) and nephrotoxicity (e.g. sodium retention, electrolyte imbalance).^13^ Their chronic use is therefore recommended to be avoided in older adults.^14^ Regarding stronger pain medication for severe pain, opioids are an option.^12^ However, their use in older adults in surgery or post-operatively is not problem-free as opioids can exacerbate delirium and have a serious risk for overdose. In addition, they can cause severe sedation-related disorders, including respiratory depression and death.^14^ Effective and proper pain medication is a crucial part of hip fracture treatment also post-operatively.^15^ It enables progressive rehabilitation needed to restore the functional capacity^1,16^ which decreases the risk for adverse effects or complications after surgery.^15^

Physical activity can reduce pain by the endogenous inhibitory system, and regular exercise increases serotonin levels which can decrease the perception of pain.^17^ In older adults’ physical activity has reduced pain and pain-related symptoms in chronic pain.^18^ There is scarce evidence of how the pain changes after the hip fracture surgery and how rehabilitation can affect pain and its interference.

These secondary analyses of a randomised controlled trial were performed to study the effects of a year-long, supervised home-based exercise programme on perceived pain, especially on pain intensity, pain interference, and the use of pain medications over 12 months after hip fracture surgery.

## Methods

These are the secondary analyses of a randomised controlled trial that was conducted in South Karelia, Finland between 2014 and 2019. Before recruitment, the trial received ethical approval from the ethics committee of the Helsinki University Hospital and was registered to ClinicalTrials.gov (NCT02305433). The protocol of the trial,^19^ and the results of the primary outcome, days lived at home,^20^ and secondary outcomes, functioning^21^ and quality of life^22^ have been published previously. The participants were recruited from the rehabilitation hospital adjacent to the operating hospital. First, the research nurse or research physiotherapist met the patient with a recently operated hip fracture and explained the trial procedures. After discharge, the research nurse or physiotherapist made a home visit and ensured eligibility to the study.

Patients were included if they had a hip fracture with International Classification of Diseases codes S72.0, S72.1 or S72.2, were aged 60 or above, were living at home (with or without home care), were able to walk indoors (walking aid allowed), had a Mini-Mental State Examination^23^ score of ≥12 points (normal cognition or mild or moderate cognitive impairment), and could speak Finnish. Patients were excluded if their life expectancy was less than two years, they were living in a 24-h nursing facility, or had a severe medical condition that prevented participation in physical exercise training (e.g. severe cardiovascular or neurological diseases), severe cognitive impairment, substance abuse, or severe hearing or eyesight problems. In 2015 two of the original inclusion criteria were modified as age was decreased from 65 to 60, and the cognition score from 17 to 12. The criteria were modified to intensify recruitment, but this had no marked impact.

If the patient was eligible and willing, baseline assessments were scheduled. Before the baseline assessments, the patient signed an informed consent document. If the patient had moderate cognitive impairment also the next of kin was present when they signed the consent form.

After the baseline assessments, the participants were allocated at one-to-one ratio to Physical Exercise and Usual Care groups by one of the research group members who had not met the participant. Randomisation was performed with a computer-generated, random sequence allocation programme, which included varying block sizes from 2 to 10. The programme was created by a statistician who did not participate in this trial.

After the hip fracture, both groups received care according to the local hip fracture care policies and national guidelines (Table 1). Both groups were allowed to use any social and health care services they needed and were entitled to, including rehabilitation. Physical Exercise group participated in physiotherapist-led exercise sessions twice a week for 60 minutes for 12 months, at their home. Exercise sessions included warm-up, strength, balance, functional, and flexibility exercises. The exercise intervention was tailored to the participant's health and fitness status. The progression of strength training was ensured with multiple-repetition maximum tests, which were performed monthly to evaluate appropriate resistance for the main exercises. The intensity of exercise sessions was evaluated with Ratings of Perceived Exertion scale^24^ with the target of moderate (12) to vigorous (17). Strength and balance exercises were based on the Otago exercise programme.^25^ Resistance was added with ankle weights, dumbbells, and weight vests.

**Table 1.** Contents of Usual care and Home-based physical exercise interventions in template for intervention description and replication format.

	Usual care	Home-based physical exercise
Why	To provide the support needed to recover from the hip fracture surgery, according to Finnish national guidelines and local policies on hip fractures	Usual care + long-term exercise training to support recovery of functioning after hip fracture surgery
What	Instructions of the movements and positions that should be avoided. Standardised training instructions after hip fracture (range of motion, strength training, etc.). At the time of discharge, the need for rehabilitation at home was evaluated. Based on this, patient had two options: Self-training with written instructions Physiotherapist supervised home-based rehabilitation (about 2 times a week, max. 4 weeks), followed by self-training with written instructions	Same materials and procedures as in Usual Care + Physiotherapist supervised home-based training for one hour, twice a week for 12 months. Main exercises strength, balance, functional, and flexibility exercises based on Otago-program^25^
Who provided	Physiotherapists of the South Karelia social and health care district	Interventions provided by physiotherapists from the seven local physiotherapy companies, selected by a procurement process and trained to provide the 12-month intervention. Physiotherapists, reported monthly the number of visits, contents, adverse effects to the study coordinator
How	Face to face individual, instruction sheets for self-training	Face to face, individual
Where	At the rehabilitation hospital, control visit at hospital, at the participants’ home	Intervention at the participants’ home
When and How Much	After discharge max. 4 weeks, 2 times a week (supervised) and unsupervised as long as needed	For 12 months after discharge twice a week for one hour under physiotherapist-supervision
Tailoring	Based on needs, mainly standardised but if needed also tailored	Tailored for the participants’ needs and health status, main characteristics were standardised
Modifications	No modifications during the trial	No modifications during the trial
How well	Per person year, participants received mean 18 rehabilitation visits (SE 3)	Per person year, participants received mean 95 (SE 3) rehabilitation visits

Participants were assessed four times: at baseline, and after 3, 6 and 12 months. A research nurse or a physiotherapist made a home visit, including measurements, interviews and questionnaires. Assessors were not blinded for the group allocation. As background information age, sex, mode of living, education and marital status were enquired. Information on the use of pain medications was queried by interviewing, checking prescription lists, medication packages, and when needed supplementing with information from electronic patient records.

In addition, morbidities were queried and confirmed from the electronic medical records. The Charlson Comorbidity Index^26^ was calculated. The participants also answered to the 15-question version of the Geriatric Depression Scale.^27^ Information on the hip fracture and its surgery was acquired from medical records.

Global pain intensity was assessed with Visual Analogue Scale.^28^ The participant marked the intensity of the pain on the 100-mm pain intensity line on paper. The place where the pain occurred was queried by listing parts of the body. Pain interference was assessed with a question based on the Short-Form health survey,^29^ ‘How much does the pain interfere with your normal activities of daily living?’. The question had six answering options: No pain / not at all (coded as 0%), a little bit (25%), moderately (50%), quite a bit (75%), and extremely (100%). Options were coded as a percentage of interference similarly to Short-Form health survey.^29^

All analyses were performed according to the intent-to-treat principle. Descriptive statistics of the participants at baseline are presented as means with Standard Deviations, medians with interquartile ranges or as frequencies with percentages (%). The intensity of pain at four time points (baseline, 3, 6, and 12 months) was analysed using a Generalised Estimating Equations model. Pain interference at the four assessment points was analysed using mixed-effects models, with an unstructured covariance structure, and the degrees of freedom were calculated by the Kenward-Roger method. The fixed effects were group, time, and group-time interactions, using age and sex as covariates. All available data were analysed with the full analysis set as mixed models enabled analyses of unbalanced datasets without imputation. All analyses were adjusted by age and sex, as the groups differed at baseline. Effect sizes (Cohen's *d*) were calculated to determine the magnitude of the difference in changes over 12 months between the groups. Effect size for changes in proportions between groups over time in prevalence of pain and pain medication users were estimated using logistic regression including interaction terms (age, sex). To convert the odds ratios to effect sizes, we applied a formula: natural logarithm of the odds ratio divided by 1.81, which approximates Cohen's *d*.^30^ Effect size values of 0.20, 0.50, and 0.80 indicate small, moderate, and large, respectively. Statistical analyses were performed using Stata 17.0, StataCorp LP (College Station, Texas, United States of America) statistical package by statistician who was blind to group allocation.

Power calculation was performed for the primary outcome, days lived at home.^19^ In brief, a sample size of 99 people was needed in each group (simulation-based effect size was 0.40) to detect a difference (alpha) 0.05, (power) 80% of the hypothesised 180 (SD 431) days in the primary outcome between the usual care and physical exercise groups.

## Results

During recruitment from December 2014 to the end of 2019, in total 541 persons underwent surgical repair of hip fracture in South Karelia County in Finland. Of them, 338 were screened as preliminary eligible and were contacted in the rehabilitation hospital, and of those the research nurse or physiotherapist met 144 patients at their homes and verified their eligibility for the study. Altogether 121 patients were eligible and willing to participate, and they were randomised to Physical Exercise group (*n* = 61) and Usual Care group (*n* = 60). The mean age of the participants was 81 (SD 7) years, and 61% (*n* = 74) had a fracture in the femoral neck. Of the participants 75% (*n* = 91) were women. As the groups differed at baseline in age and sex (Table 2), further analyses were adjusted accordingly. In the exercise group the median number for completed physical exercise sessions was 96 (IQR 88,98).

**Table 2.** Baseline characteristics in Usual Care (*n* = 60) and Physical Exercise (*n* = 61) groups.

Characteristics	Usual Care *n* = 60	Physical Exercise *n* = 61
Age, mean (SD)	80 (7)	83 (6)
Women, *n* (%)	41 (68)	50 (82)
Education years, mean (SD)	7.5 (2.6)	8.1 (3.2)
Living alone, *n* (%)	35 (58)	37 (61)
Mini-mental state examination, mean (SD)	22.7 (4.2)	23.1 (4.7)
Geriatric Depression Scale, mean (SD)	4.1 (2.9)	4.6 (2.2)
Physician-diagnosed diseases or disorders, n (%)		
Musculoskeletal complaints*	47 (78)	54 (88)
Coronary heart disease	27 (45)	27 (44)
Stroke or transient ischaemic attack	19 (32)	14 (23)
Hypertension	43 (72)	44 (72)
Diabetes	12 (20)	16 (26)
Alzheimer's disease	10 (17)	11 (18)
Charlson Comorbidity Index, *n* (%)	0.4 (0.8)	0.4 (0.6)
Fracture type (International Classification of Diseases codes), *n* (%)		
Femoral neck (S72.0)	35 (58)	39 (64)
Pertrochanteric (S72.1)	21 (35)	17 (28)
Subtrochanteric (S72.2)	4 (7)	5 (8)
Type of surgery, *n* (%)		
Total hip arthroplasty	0 (0)	2 (3)
Hemiarthroplasty	36 (59)	31 (52)
Internal fixation	25 (41)	27 (45)
Days from surgery to discharge, median (interquartile range)	25 (20; 31)	25 (19; 32)
Days from discharge to baseline assessments, median (interquartile range)	9 (6; 13)	8 (5; 12)
Pain in some parts of the body, *n* (%)	43 (72)	46 (75)
Pain in hip	40 (67)	38 (62)
Pain in other parts of the body	30 (50)	26 (43)
Number of regular medications, mean (SD)	8.7 (3.0)	8.8 (3.4)
Prescribed pain medications, *n* (%)	58 (97)	60 (98)
Non-steroidal anti-inflammatory drugs	5 (8)	3 (5)
Paracetamol	58 (97)	58 (95)
Opioids	21 (35)	20 (33)
Mild	1 (2)	2 (3)
Moderate	2 (3)	0 (0)
Strong	20 (33)	18 (30)
Combinations of different pain medications, *n* (%)		
Non-steroidal anti-inflammatory drugs + paracetamol	5 (8)	2 (3)
Non-steroidal anti-inflammatory drugs + opioid	0 (0)	0 (0)
Paracetamol + opioid	21 (35)	17 (28)
Non-steroidal anti-inflammatory drugs + opioid + paracetamol	1 (2)	0 (0)

Note: *Includes: musculoskeletal complaints: such as back pain, shoulder and neck pain, osteoarthritis, rheumatoid arthritis.

**Figure 1.**
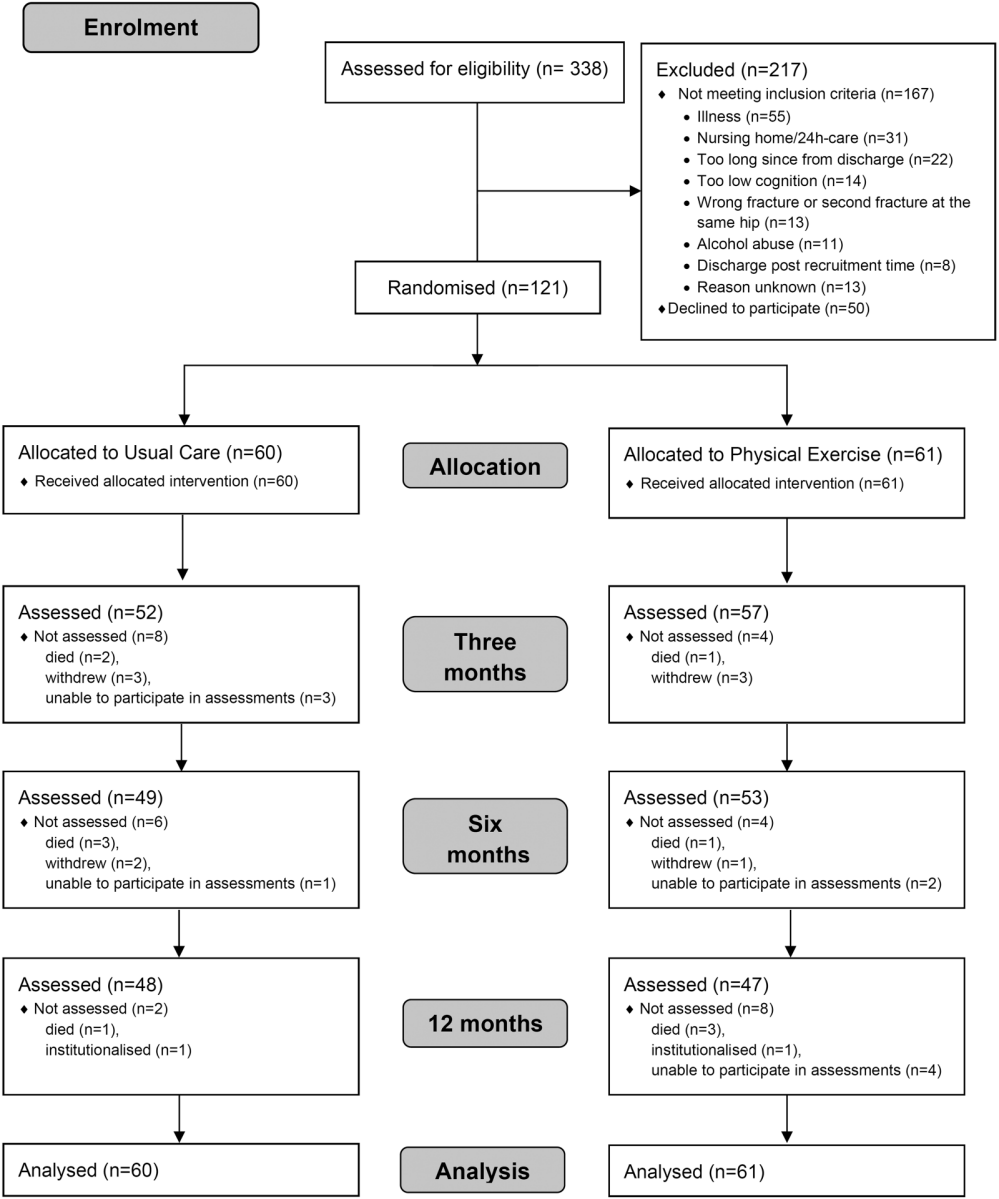
Flowchart of the study.

At 3, 6 and 12 months a total of 52, 49 and 48 participants in Usual Care and 57, 53 and 47 participants in Physical Exercise group were assessed, respectively (Figure 1).

**Figure 2.**
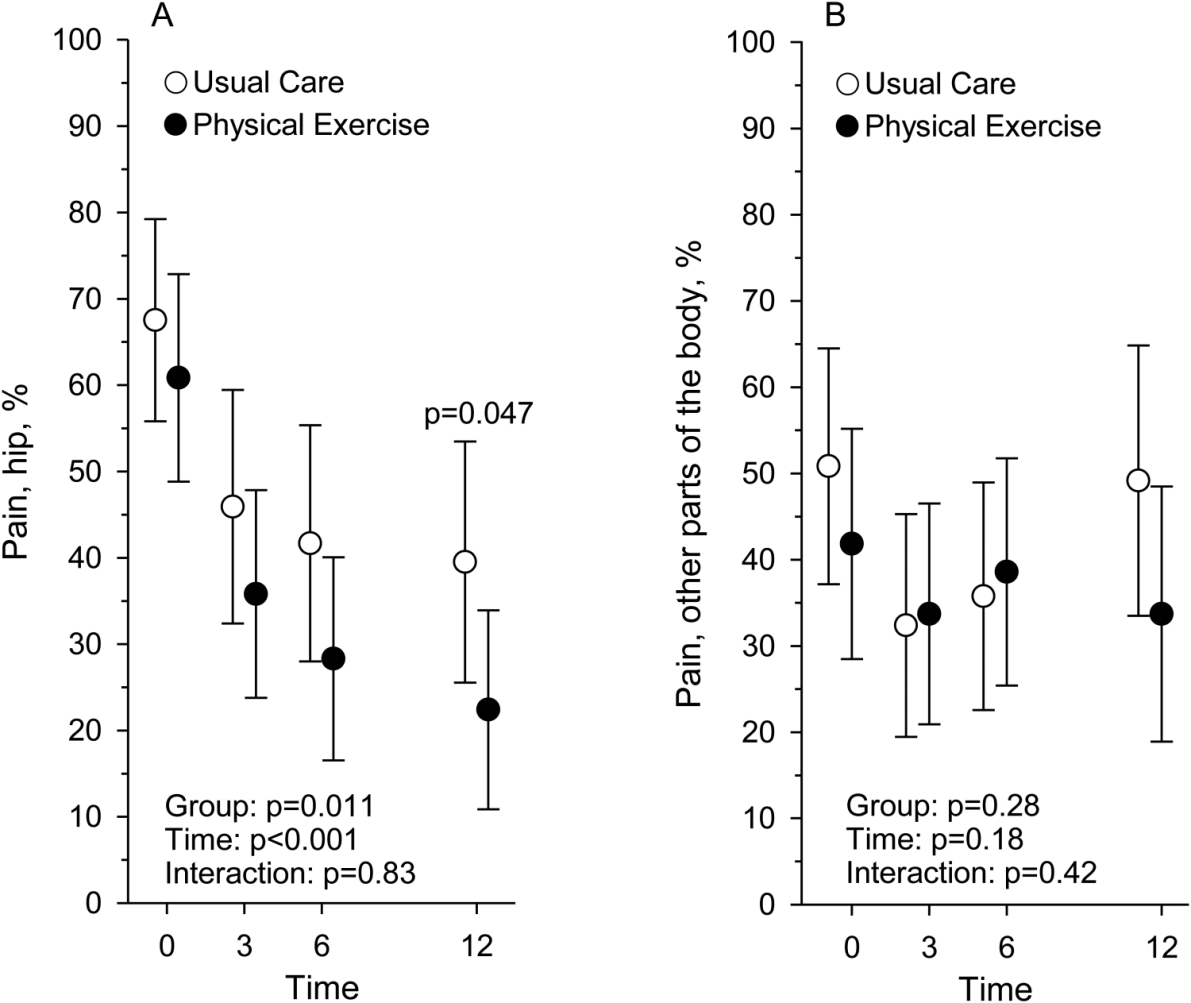
The proportion (%) of participants reporting pain in the hip region (a) and in other parts of the body (b) at baseline and at 3, 6, and 12-month assessment points in Usual Care (*n* = 60) and Physical Exercise (*n* = 61) groups.

At baseline (Table 2), 43 (72%) participants in Usual Care and 46 (75%) participants in Physical Exercise reported pain in some parts of their body. Hip pain was the most common, as 40 (67%) participants in Usual Care and 38 (62%) in Physical Exercise reported it (Table 2, Figure 2(a)).

At 12 months the proportion of participants reporting hip pain was significantly lower than at baseline, and the prevalence of hip pain was significantly lower in Physical Exercise than in Usual Care (*P* = .047), as 18 (38%) participants in Usual Care and 11 (23%) in Physical Exercise reported hip pain (Figure 2(a), Table 3). At 12 months, there was no difference (*P* = .61) between the groups in the prevalence of pain in other parts of the body, as the number of participants reporting pain was 31 (65%) in Usual Care and 28 (60%) in Physical Exercise (Figure 2(b), Table 3).

**Figure 3.**
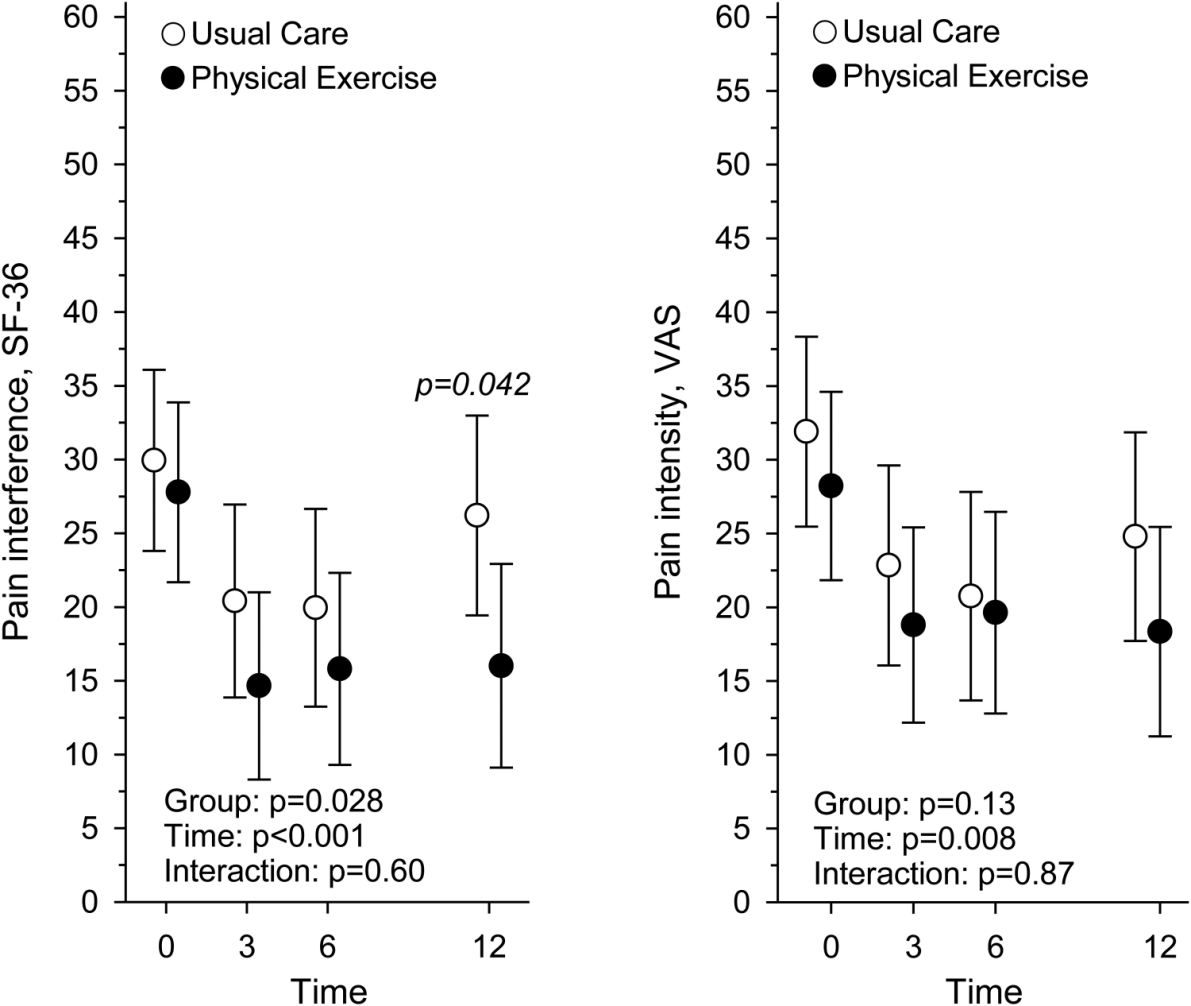
Pain interference measured with Short-Form health survey, and pain intensity measured with visual analogue scale, at baseline and at 3, 6, and 12-month assessment points in Usual Care (*n* = 60) and Physical Exercise (*n* = 61) groups.

The interference of pain (Figure 3) and pain intensity (Figure 3) were the highest at baseline and decreased in both groups over 12 months (Table 3). At 12 months, the participants in Physical Exercise reported less pain interference than those in Usual Care (*P* = .042). Pain intensity decreased over 12 months and there was no difference between the groups in pain intensity at 12 months.

**Table 3.** Pain (location, interference and intensity) and pain medication at baseline, their changes over 12 months and effect sizes for changes.

	Baseline		Change to 12 months^a^		Effect size (95% CI)^a^
	Usual Care *n* = 60	Physical Exercise *n* = 61	Usual Care (95% CI)	Physical Exercise (95% CI)	
Participants reporting pain, *n* (%)					
Hip	40 (67)	38 (62)	−30 (−47 to −12)	−39 (−56 to −22)	−0.38 (−0.51 to −0.22)
Other	30 (50)	26 (43)	−2 (−19 to 16)	−8 (−25 to 10)	−0.26 (−0.70 to 0.17)
Pain, mean (SD)					
Interference	30 (29)	28 (22)	−5 (−14 to 4)	−12 (−21 to −3)	−0.18 (−0.52 to −0.05)
Intensity	32 (29)	28 (24)	−8 (−18 to 1)	−10 (−19 to −1)	0.01 (−0.45 to 0.47)
Users of pain medications, *n* (%)					
Any pain medications	58 (97)	60 (98)	−10 (−20 to −1)	−6 (−14 to 3)	0.12 (−0.67 to 0.91)
Opioids	21 (35)	20 (33)	−25 (−40 to −11)	−17 (−33 to −2)	0.24 (−0.47 to 0.95)

^a^ Adjusted for age and sex.

**Figure 4.**
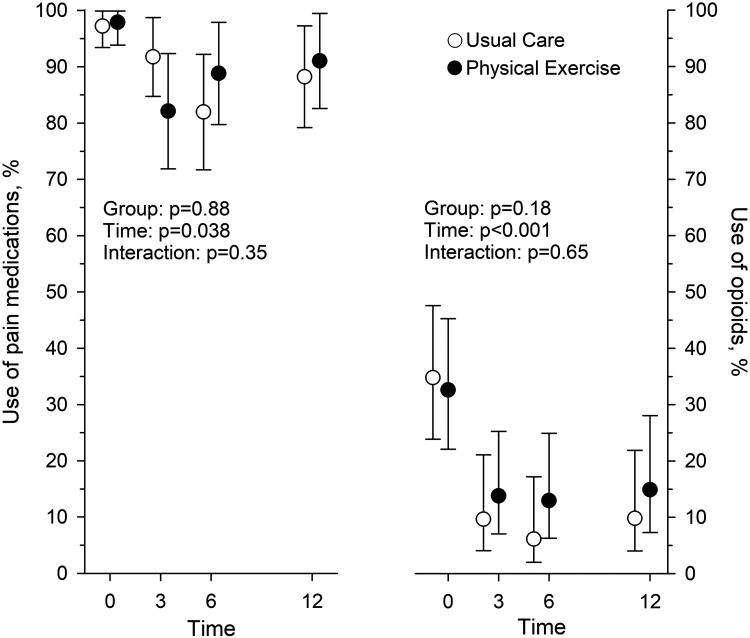
The proportion (%) of participants using pain medication and of those using opioids baseline and at 3, 6, and 12-month assessment points in Usual Care (*n* = 60) and Physical Exercise (*n* = 61) groups.

At baseline 118 (98%) of the participants used prescribed pain medications (Table 2). The most common pain medication was paracetamol, which was used by 58 (97%) participants in Usual Care and by 58 (95%) participants in Physical Exercise. Opioids were used by 21 (35%) and 20 (33%) participants, respectively. There was no difference between Usual Care and Physical Exercise groups in the proportion of users of any pain medications or in those who used opioids in any assessment point (Figure 4, Table 3). In both groups the number of users of opioids decreased to 3 months and stayed at that level at 6 and 12 months (Figure 4).

## Discussion

One year since hospital discharge of hip fracture surgery, the proportion of participants reporting hip pain was significantly lower in those who participated in home-based physical exercise training than in those in usual care. In addition, the participants in Physical Exercise group reported less pain interference at 12 months compared to Usual Care. The use of opioids and other pain medications decreased in both groups during the year and there was no difference between the groups in the number of users of pain medication over the 12 months.

Our participants were discharged from the rehabilitation hospital on average 30 days after the surgery, and the baseline measurements were performed within 2 weeks since discharge. In our trial, most of the participants in both groups had femoral neck fractures corrected with hemiarthroplasty. The type of surgery and fixation method chosen can affect perceived pain and mobility, as the patients with internal fixations tends to report more intense pain and walking difficulties compared to hemiarthroplasty patients.^31^

Our Physical Exercise group reported less pain interference in their activities of daily living at 12 months compared to those in usual care. As previously reported Physical Exercise group also had more functional independence^20^ and better functional capacity^21^ at 12 months than Usual Care group. Proper pain management after discharge enabled the rehabilitation at home, which increases the probability of functional capacity to recover back to the pre-surgery level.^1,16^ Previous studies have shown that the postoperative pain can be managed with pain medications, but also with non-pharmacological interventions, for example physical therapy techniques such as cryotherapy (cold therapy), transcutaneous electrical nerve stimulation.^32^

As postoperative pain is associated with long-term functional impairment, administering appropriate opioid analgesics post-operatively can enhance the recovery of functional capacity as it allows earlier ambulation.^33^ Functional recovery is also associated with the type of surgery, surgeon's technique, postoperative complications, pain, hospitalisation time, and post-operative care.^34^ At baseline, opioids were used by 41 participants (34%) and paracetamol by 116 (96%) as prescribed by their physician at discharge. During the 12 months since discharge the use of pain medications did not change significantly, but the use of opioids decreased over 12 months, especially during the first 3 months. At 12 months about 10–15% still used opioids. In a Danish cohort study, 62% of the patients with hip fracture were using opioids at discharge, and after one year 30% of those were still using them.^35^ In another study, opioids were prescribed for 54% of the patients discharged from inpatient rehabilitation facilities and for 22% discharged from skilled nursing facilities.^36^ Even though prescribing opioids after hip fracture at discharge from the post-acute rehabilitation facility is common, it is important to gradually decrease the opioids when they are no longer clinically indicated, to avoid adverse outcomes and addiction.^37^

The prolonged rehabilitation after the hip fracture surgery is widely studied,^1,15,16^ but there are not many studies that have reported the effects on pain or pain medication usage over 12 months. Pain after hip fracture is associated with an inactive lifestyle^38^ and difficulties with the task related to activities of daily living and declined quality of life.^39^ In general, physical exercise training has been shown to reduce pain perception, chronic pain and pain-related symptoms in older adults.^17,18^ In a Korean study the 8 weeks of multicomponent home rehabilitation after hip fracture surgery reduced pain.^40^ In another study, older adults with hip fracture history reported more pain in the lower limbs in general compared to non-fracture age-matched controls even several years after the surgery.^31^

The strengths of this study are that it is a rigorously performed randomised controlled trial with good compliance, as the median number of home-based exercise sessions was 96 of the possible 104. Our home-based physiotherapist-supervised physical exercise training was also well tolerated, without adverse effects and the participants reported only mild exercise-related muscle soreness without any complications to the hip.^20^ Pain medication usage was queried from the participants and information was complemented with the information retrieved from the electronic patient records, which increases the reliability of the information.

Limitations of this study concern the assessment of pain intensity and interference. The participants were asked to report body parts where they felt pain at the time of the assessments, but the intensity and interference referred to global pain, not specifically for each body part. Pain in multiple sites can increase the risk of fractures^41^ and the use of opioids can cause falls which lead to fractures.^42^ We did not have information on pain medications used before hip fracture, nor information on previous pain and its location or intensities, which is a shortcoming. This article represents the results of the secondary analyses of the randomised controlled trial, which was designed to find a difference between groups in the days lived at home rather than medication use or pain-related outcomes.

The year-long twice-a-week physiotherapist-supervised home-based physical exercise training regimen reduced prevalence of hip pain and pain interference compared to usual care. Over 12 months the number of users of pain medication decreased, with no difference between the groups.

**Clinical messages**

- Prolonged rehabilitation after the hip fracture surgery, with physiotherapist supervised home-based physical exercise twice a week for 12 months, contributes to less pain in the hip area and less pain interference compared to Usual Care.
- The use of pain medication decreased in both groups (Physical Exercise, Usual Care) over the 12 months after hip fracture surgery. The use of pain medications, (including opioids) did not differ among participants in 12-month supervised home-based physical exercise and Usual Care.
- Pain medication is a crucial part of recovery from hip fracture, and 12-month twice a week physical activity did not increase the need for pain medication compared to Usual Care.
- This Randomised Clinical Trial supports the use of long-term physiotherapist-supervised home-based exercise as part of post-hip fracture rehabilitation as it is well tolerated among older adults.
